# Synthetic-to-Clinical Ensemble Learning for Volumetric Breast Tumor Segmentation in Digital Breast Tomosynthesis Under Limited Annotated Data

**DOI:** 10.3390/diagnostics16183046

**Published:** 2026-09-20

**Authors:** Cristina Alfaro, Gabriel Guerra, Claudia Prieto, Domingo Mery

**Affiliations:** 1 Department of Medical Technology, Facultad de Ciencias de la Salud, Universidad de Tarapacá, Arica 1000000, Chile; 2 Institute for Biological and Medical Engineering, Pontificia Universidad Católica de Chile, Santiago 7820436, Chile; 3 PhD Program in Health Sciences and Engineering, Universidad de Valparaíso, Valparaíso 2340000, Chile; gabriel.guerrap@postgrado.uv.cl; 4 Millennium Institute for Intelligent Healthcare Engineering iHealth, Santiago 7820436, Chile; cdprieto@uc.cl (C.P.); domingo.mery@uc.cl (D.M.); 5 Department of Electrical Engineering, School of Engineering, Pontificia Universidad Católica de Chile, Santiago 7820436, Chile; 6 Department of Computer Science, School of Engineering, Pontificia Universidad Católica de Chile, Santiago 7820436, Chile

**Keywords:** digital breast tomosynthesis, breast tumor segmentation, breast cancer, deep learning, synthetic data, synthetic-to-clinical transfer, transfer learning, ensemble learning

## Abstract

**Background/Objectives**: Digital breast tomosynthesis (DBT) provides quasi-three-dimensional breast imaging, but volumetric segmentation model development is constrained by limited expert-annotated clinical data. This proof-of-concept study evaluated the feasibility of synthetic-to-clinical breast tumor segmentation using synthetic model development, limited clinical fine-tuning, and heterogeneous ensemble learning, and aimed to establish a reproducible low-resource framework for volumetric DBT segmentation. **Methods**: Three architectures—3D U-Net, nnU-Net, and Attention U-Net—were evaluated using publicly available synthetic and clinical DBT datasets. Models initialized from the Mixed-size synthetic configuration were fine-tuned using 10 clinical development cases with five-fold cross-validation; 10 additional clinical cases were reserved for independent testing. Ensemble weights and threshold were evaluated from development-set out-of-fold predictions. Performance was assessed using Dice, Intersection-over-Union (IoU), precision, and recall, with paired non-parametric comparisons on the clinical test cohort. **Results**: nnU-Net achieved the highest mean Dice on the Large Tumor (0.864) and Mixed-size (0.841) synthetic test sets, while performance was lower in the Small Tumor configuration (0.560). On the independent clinical cohort, the ensemble achieved the highest mean Dice (0.518) and recall (0.630), compared with nnU-Net (Dice = 0.482), Attention U-Net (0.372), and 3D U-Net (0.367). After Holm correction, ensemble Dice was significantly higher than Attention U-Net and 3D U-Net, but not nnU-Net. **Conclusions**: Synthetic DBT data provided a useful source-domain foundation for volumetric segmentation under constrained annotation conditions, although a synthetic-to-clinical gap remained. Heterogeneous ensemble integration achieved the highest mean clinical Dice without demonstrating superiority over fine-tuned nnU-Net. Larger and more diverse clinical cohorts are required to establish generalizability.

## 1. Introduction

Digital breast tomosynthesis (DBT) has emerged as a major advancement in breast imaging by providing quasi-three-dimensional visualizations of breast tissue, thereby reducing the masking effects of tissue superposition commonly encountered in conventional digital mammography [1,2]. Compared with conventional mammography, DBT improves lesion conspicuity and diagnostic confidence, particularly in women with dense breast tissue. However, the increased volumetric information substantially increases the complexity of image interpretation, requiring radiologists to review numerous reconstructed slices for each examination, thereby increasing reading time and cognitive workload in routine clinical practice [3,4,5]. These challenges have stimulated growing interest in artificial intelligence (AI) methods capable of assisting radiologists through automated image analysis and quantitative characterization of breast lesions.

Recent advances in deep learning have transformed medical image analysis, achieving remarkable performance in lesion detection, classification, and semantic segmentation across multiple imaging modalities [6]. In breast imaging, however, most AI studies have focused on two-dimensional mammography, ultrasound, or breast magnetic resonance imaging (MRI), whereas comparatively fewer investigations have addressed volumetric DBT analysis [7,8,9]. Existing DBT studies have primarily concentrated on lesion detection, breast density estimation, imaging system evaluation, or slice-based analyses rather than complete volumetric tumor segmentation [10,11,12,13,14]. However, accurate three-dimensional tumor delineation is clinically relevant because it enables quantitative characterization of lesion morphology and extent, facilitates radiomic analysis, and may support treatment planning and longitudinal therapy assessment, as has been demonstrated in breast MRI and other fully volumetric imaging modalities [15,16,17,18]. Nevertheless, extending these capabilities to DBT remains challenging due to the modality’s limited-angle acquisition geometry, anisotropic spatial resolution, reconstruction artifacts, and reduced lesion conspicuity compared with fully tomographic imaging [19,20].

A major obstacle to developing robust deep learning models for volumetric DBT segmentation is the limited availability of annotated three-dimensional clinical datasets. Producing voxel-level reference annotations is labor-intensive, requires specialized radiological expertise, and remains difficult to scale in routine clinical practice [21,22,23]. These limitations are particularly critical under low-resource research conditions, where only small collections of expert-annotated DBT examinations are available for algorithm development and validation. Consequently, many published segmentation studies have relied on lesion-centered regions of interest (ROIs), cropped image patches, or relatively small institutional datasets to reduce computational complexity and annotation burden [24,25,26,27]. While these strategies have facilitated methodological development, they implicitly simplify the segmentation problem by constraining the search space and providing prior lesion localization, limiting their resemblance to real clinical imaging workflows.

To address the scarcity of annotated clinical data, synthetic medical imaging has emerged as a promising strategy for supporting reproducible algorithm development while simultaneously overcoming privacy and data-sharing constraints [28,29,30,31,32]. In DBT, physics-based simulation frameworks employing anthropomorphic digital breast phantoms enable the generation of anatomically realistic breast volumes together with complete voxel-level ground-truth annotations under fully controlled imaging conditions [33,34]. These synthetic datasets allow systematic variation in clinically relevant factors, including breast density, lesion morphology, lesion size, and acquisition parameters, providing an attractive platform for developing and benchmarking deep learning algorithms. More recently, synthetic datasets have also been investigated as source domains for pretraining deep learning models before adaptation to limited clinical datasets, to reduce the dependence on large annotated clinical cohorts while improving model robustness under constrained data conditions [35,36].

Despite these advantages, an important challenge remains unresolved: whether representations learned from synthetic DBT data can effectively generalize to real clinical examinations. The transition from synthetic to clinical imaging introduces a substantial domain shift arising from differences in anatomical variability, tissue heterogeneity, acquisition protocols, reconstruction algorithms, imaging noise, and patient-specific characteristics [37,38,39]. In this context, improving synthetic-to-clinical transfer has been an active area of research in medical image analysis, where transfer learning, domain adaptation, and fine-tuning strategies are increasingly explored to enhance generalization when only limited target-domain data are available [35,40].

Beyond data availability, increasing evidence suggests that model diversity represents an additional factor influencing segmentation robustness. Different deep learning architectures frequently learn complementary feature representations and exhibit distinct strengths and failure modes depending on their architectural design, optimization strategy, and feature extraction mechanisms. Consequently, relying on a single architecture may limit robustness when models are exposed to heterogeneous clinical data. Ensemble learning addresses this limitation by integrating complementary predictions from multiple independently optimized models, thereby reducing architecture-specific prediction errors and improving robustness, reliability, and generalization under distributional shifts. This principle has been successfully demonstrated in heterogeneous ensembles for brain tumor segmentation, complementary deep learning ensembles for breast lesion segmentation in dynamic contrast-enhanced MRI, and ensemble-based strategies for breast MRI tumor delineation exploiting complementary network predictions [41,42,43]. Collectively, these studies indicate that complementary model representations may improve segmentation robustness beyond the capabilities of individual architectures. The combined use of synthetic DBT pretraining, limited clinical adaptation, and heterogeneous ensemble integration remains comparatively underexplored for volumetric DBT tumor segmentation. Consequently, whether complementary model representations can improve synthetic-to-clinical generalization in DBT remains an open research question.

To contextualize the methodological position of the present study, Table 1 summarizes representative previous investigations relevant to DBT lesion analysis and volumetric medical image segmentation. The comparison highlights differences in imaging modality, input formulation, segmentation strategy, data source, and clinical adaptation. Rather than providing a direct performance ranking, the table is intended to situate the present synthetic-to-clinical framework within the broader methodological landscape.

Motivated by these considerations, this proof-of-concept study investigates the feasibility of synthetic-to-clinical volumetric breast tumor segmentation in digital breast tomosynthesis under limited annotated data conditions. Unlike approaches based primarily on lesion-centered or region-of-interest inputs, the proposed framework evaluates segmentation using 15-slice volumetric inputs that preserve the complete in-plane breast field of view and integrates synthetic model development, limited supervised clinical adaptation, and heterogeneous ensemble learning. The study aims to (1) characterize segmentation performance across synthetic datasets with different lesion-size distributions, (2) evaluate synthetic-to-clinical transfer under limited clinical supervision, and (3) determine whether heterogeneous ensemble integration can improve clinical segmentation performance on an independent held-out DBT cohort.

The main contributions of this work are: (i) a reproducible synthetic-to-clinical framework for volumetric DBT tumor segmentation under constrained clinical annotation conditions; (ii) a systematic evaluation of three established volumetric segmentation architectures across synthetic, Hybrid synthetic–clinical, and sequential clinical fine-tuning strategies; (iii) a heterogeneous ensemble framework developed exclusively from clinical development-set predictions, including systematic weight–threshold ablation and stability analyses; and (iv) an independent clinical evaluation incorporating case-level uncertainty estimates, paired statistical comparisons, and qualitative assessment of challenging segmentation outcomes.

## 2. Materials and Methods

### 2.1. Study Design

This proof-of-concept study investigated synthetic-to-clinical volumetric breast tumor segmentation in DBT under limited annotated data conditions, defined by the restricted availability of expert-annotated clinical DBT examinations for model development and evaluation. A total of 20 annotated clinical DBT examinations were available. Ten cases were assigned to the clinical development cohort for model adaptation and ensemble development, whereas the remaining 10 cases were reserved as an independent clinical test cohort and were excluded from model-development decisions.

The experimental framework was designed to evaluate three complementary components of synthetic-to-clinical learning: synthetic-domain model development, limited supervised clinical adaptation, and heterogeneous ensemble integration. Three volumetric segmentation architectures were evaluated: a baseline 3D U-Net [46], nnU-Net [47,48], and Attention U-Net [49].

The study comprised two related experimental pathways using the dataset configurations described in Section 2.2. In the first pathway, the three architectures were evaluated under synthetic-only training conditions and in a separate one-stage configuration combining synthetic data with the clinical development cohort. In the second pathway, models trained on the designated synthetic source configuration were subsequently adapted using the clinical development cohort and combined through heterogeneous ensemble learning. Model development and ensemble configuration procedures were confined to the corresponding training and development data, whereas the independent clinical test cohort was reserved for final performance evaluation.

**Figure 1:**
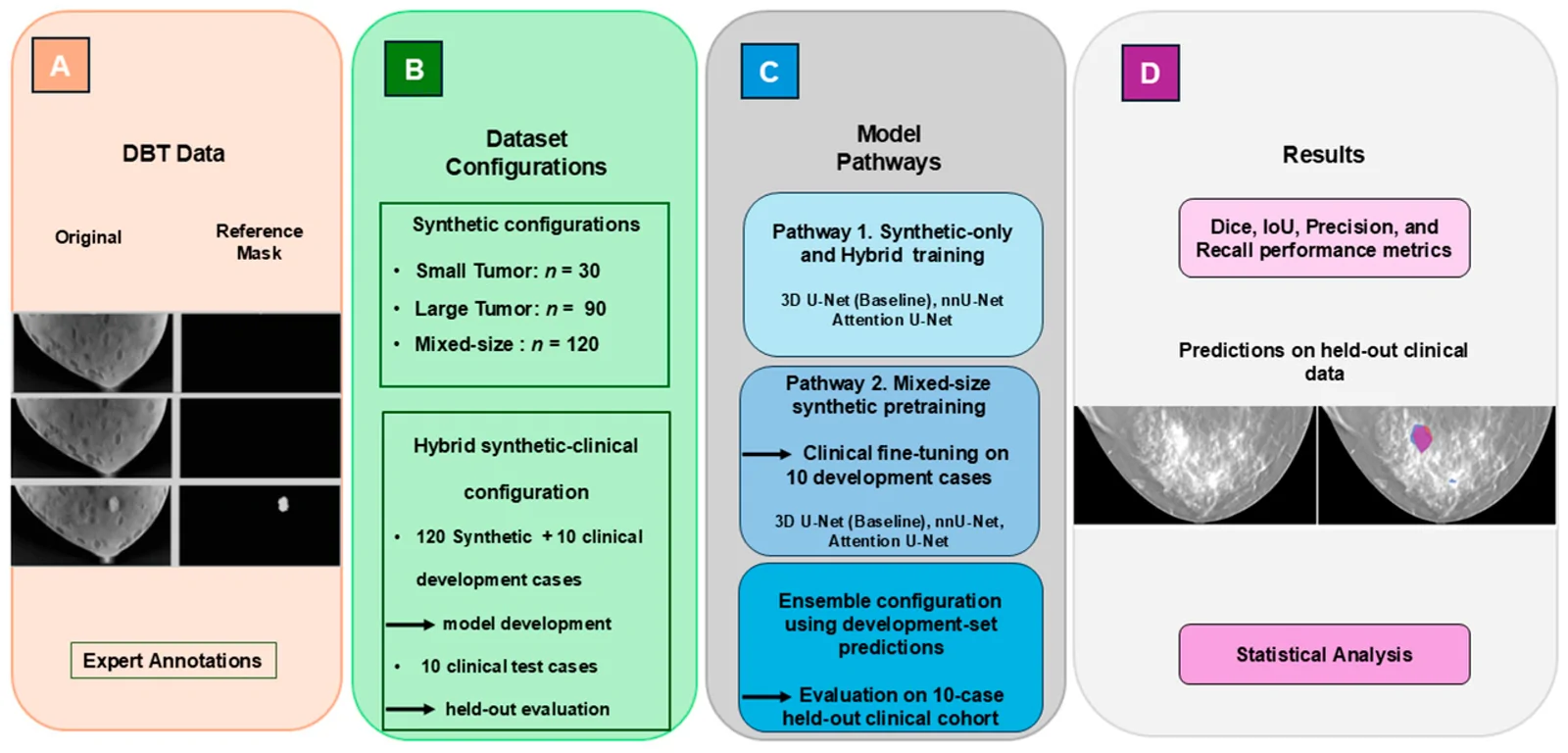
Overview of the experimental framework. (**A**) Representative DBT data and corresponding expert reference masks. (**B**) Dataset configurations comprising the Small Tumor (*n* = 30), Large Tumor (*n* = 90), and Mixed-size (*n* = 120) synthetic cohorts, together with the separate Hybrid synthetic–clinical configuration, in which 120 synthetic cases and 10 clinical development cases were used for model development while 10 additional clinical cases were reserved for held-out evaluation. (**C**) Two experimental model pathways involving 3D U-Net, nnU-Net, and Attention U-Net: (i) independent synthetic-only and Hybrid training and (ii) Mixed-size synthetic pretraining followed by clinical fine-tuning using the 10-case development cohort, development-set ensemble configuration, and evaluation on the held-out clinical cohort. (**D**) Quantitative segmentation metrics, qualitative predictions, and statistical analysis.

All experiments used 15-slice volumetric inputs while preserving the full in-plane breast field of view; no lesion-centered in-plane cropping was performed. The study was not intended to establish a clinical performance benchmark or identify a universally superior segmentation architecture, but rather to evaluate the methodological feasibility of synthetic-to-clinical learning under constrained clinical annotation conditions. An overview of the experimental framework is shown in Figure 1.

### 2.2. DBT Datasets and Experimental Configurations

The study used publicly available synthetic and clinical DBT datasets representing complementary imaging domains. The synthetic datasets provided controlled lesion characteristics, whereas the clinical dataset provided real-world imaging variability; reference tumor masks were handled according to the unified expert annotation and review protocol described in Section 2.3.

The synthetic source domain comprised 30 DBT cases from the BreasTomo-Synth Version 1.1 dataset [33], containing small lesions ranging from 3 to <5 mm, and 90 DBT cases from the T-Synth dataset [34], containing larger lesions ranging from >5 to 7 mm. Both datasets were generated using simulation methodologies derived from the VICTRE framework described by Badano et al. [30,50], thereby providing a methodologically consistent synthetic source domain for model development.

The clinical data comprised 20 DBT examinations containing malignant, biopsy-proven tumors from the publicly available BCS-DBT dataset [21]. Available public metadata did not provide maximum tumor diameter, T stage, or neoadjuvant treatment history. The selected examinations represented single-lesion cases; more detailed multifocal or multicentric characterization was unavailable. Before model development, these cases were divided into two mutually exclusive subsets: 10 clinical development cases and 10 independent clinical test cases. The development cases were used exclusively for clinical adaptation and ensemble-development procedures, whereas the test cases were reserved for independent evaluation.

Four experimental dataset configurations were considered: (i) the Small Tumor synthetic dataset (*n* = 30); (ii) the Large Tumor synthetic dataset (*n* = 90); (iii) the Mixed-size synthetic dataset, combining the two synthetic lesion-size groups (*n* = 120); and (iv) the Hybrid synthetic–clinical configuration (*n* = 140). For the Hybrid experiment, the 120 synthetic cases were combined with the 10 clinical development cases for model development, while the fixed 10-case clinical test cohort remained exclusively reserved for evaluation. The Hybrid configuration was evaluated as a separate one-stage synthetic–clinical training strategy and did not provide the pretrained weights used for the subsequent clinical fine-tuning pathway.

### 2.3. Unified Annotation Protocol

To promote consistency across the synthetic and clinical data sources, all reference tumor masks used for model development and evaluation were subjected to a unified expert annotation and review protocol. For each DBT examination, a breast imaging expert with more than 20 years of experience first identified the lesion-containing region within the reconstructed image stack. Tumor boundaries were then manually delineated or reviewed at the voxel level using ImageJ/Fiji Version 1.54f [51].

The same boundary-definition criteria were applied across dataset configurations to provide internally consistent reference segmentations and reduce variability arising from heterogeneous labeling criteria. The use of a single expert observer was intended to maintain annotation consistency during this proof-of-concept study.

### 2.4. Data Representation and Preprocessing

Original DBT examinations, stored as stacks of reconstructed slices, were converted into three-dimensional arrays for model training and evaluation. Following expert identification of the lesion-containing region, a window of 15 consecutive reconstructed slices was selected to encompass the lesion and adjacent breast tissue. The lesion was not required to occupy the central position within this window and could therefore appear at different locations along the through-plane dimension. Depending on lesion extent and position, individual slices could contain a substantial portion of the tumor, only a small tumor component, or exclusively tumor-free breast tissue.

The complete in-plane breast field of view was preserved, and no lesion-centered in-plane cropping was applied. Consequently, lesion position remained variable within the X–Y plane as well as along the Z dimension of the 15-slice input, requiring the models to localize and segment the tumor within the surrounding breast anatomy rather than receiving a spatially centered lesion region of interest.

**Figure 2:**
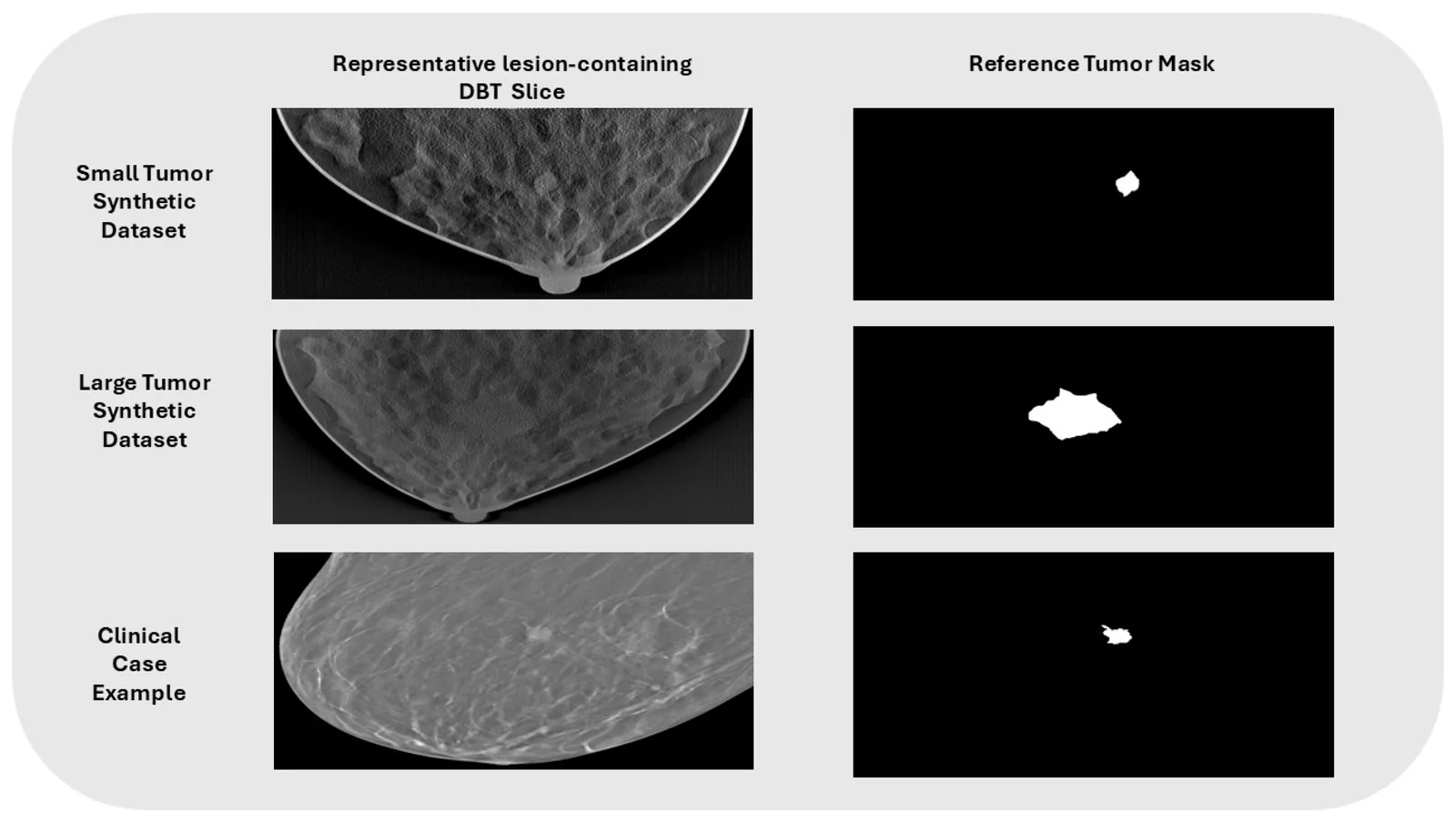
Representative lesion-containing DBT slices and corresponding reference tumor masks from the synthetic and clinical datasets. The first row shows an example from the Small Tumor synthetic dataset, the second row an example from the Large Tumor synthetic dataset, and the third row a representative clinical case from the BCS-DBT dataset. For each example, the left column shows a representative reconstructed DBT slice containing the lesion, and the right column shows the corresponding reference tumor mask. The examples illustrate the lesion-size distinction between the two synthetic configurations and differences in lesion morphology and image appearance between the synthetic and clinical imaging domains. The displayed slices should not be interpreted as the geometric center of the 15-slice model inputs.

Preprocessing included intensity clipping at the 0.5th and 99.5th percentiles, followed by z-score normalization and in-plane resizing to standardize input dimensions across datasets while preserving the 15-slice depth. Images were resized using linear interpolation, whereas reference masks were resized using nearest-neighbor interpolation and subsequently binarized. The resulting model inputs measured 512 × 256 pixels in-plane with 15 reconstructed slices (X × Y × Z: 512 × 256 × 15). The same input-preparation procedure was applied across all dataset configurations. Figure 2 shows representative synthetic and clinical DBT slices together with their corresponding reference tumor masks.

### 2.5. Network Architectures

Three volumetric convolutional segmentation approaches were evaluated: a baseline 3D U-Net, nnU-Net, and Attention U-Net. These models were selected to represent distinct but well-established strategies for three-dimensional medical image segmentation within the same synthetic-to-clinical experimental framework.

The baseline 3D U-Net [46] was included as a conventional volumetric encoder–decoder architecture. Its contracting and expanding paths, connected through skip connections, enable hierarchical three-dimensional feature extraction while preserving spatial information required for voxel-level segmentation.

The nnU-Net framework [47,48] was included as a well-established self-configuring reference approach for biomedical image segmentation. Rather than relying on a manually fixed architecture configuration, nnU-Net automatically adapts network and training-related parameters, including patch size, batch size, network depth, and anisotropic downsampling, according to the characteristics of the dataset and available computational resources.

The Attention U-Net architecture [49] incorporates attention-gated skip connections that selectively modulate encoder features before their integration into the decoder. It was included to evaluate an attention-guided volumetric segmentation strategy in which feature propagation is selectively emphasized in regions relevant to the segmentation task.

Together, these models provided three complementary methodological perspectives within the study design: a conventional volumetric encoder–decoder baseline, a self-configuring segmentation framework, and an attention-guided architecture.

### 2.6. Training Strategy and Implementation

All experiments were implemented in Python 3.10 using PyTorch (version 2.5.1) and executed on a workstation equipped with an NVIDIA GeForce RTX 4070 Ti SUPER graphics processing unit with 16 GB of memory.

For the baseline 3D U-Net and Attention U-Net, optimization was performed using AdamW [52] with an initial learning rate of 1 × 10^−3^ and a weight decay of 1 × 10^−5^. A batch size of one was used for the full-volume 3D U-Net and Attention U-Net experiments because of the volumetric memory footprint. A polynomial learning-rate decay schedule with a five-epoch warm-up phase was used, and training was conducted for up to 500 epochs using mixed-precision computation [53]. During synthetic-domain training, the baseline 3D U-Net used binary cross-entropy loss, whereas nnU-Net and Attention U-Net used combined Dice and cross-entropy loss. Loss functions used during clinical adaptation are described in Section 2.8.

We implemented nnU-Net through its self-configuring pipeline. Architecture- and resource-dependent parameters, including patch size, batch size, network depth, and anisotropic downsampling, were therefore determined automatically from the dataset characteristics and available computational resources rather than manually selected. The standard nnU-Net augmentation and foreground-aware patch-sampling procedures were retained. During inference, predictions were generated using sliding-window evaluation with 50% patch overlap and Gaussian weighting.

A fixed random seed of 42 was used throughout the experiments. Dataset partitioning was performed exclusively at the study level to prevent information leakage between training, validation, and test subsets. Complete implementation and training configurations are publicly available through the GitHub repository specified in the Code Availability statement.

### 2.7. Synthetic and Hybrid Experimental Setting

The first experimental pathway evaluated the three segmentation architectures under synthetic-only and combined synthetic–clinical training conditions. Separate experiments were conducted using the Small Tumor, Large Tumor, and Mixed-size synthetic configurations defined in Section 2.2. These experiments were used to characterize segmentation performance across different synthetic lesion-size distributions. Models were trained independently from random initialization within each configuration.

For the baseline 3D U-Net and Attention U-Net, study-level training, validation, and held-out test partitions comprised 22/5/3 cases for the Small Tumor configuration, 66/16/8 cases for the Large Tumor configuration, and 87/22/11 cases for the Mixed-size configuration, respectively. The same held-out test cases within each synthetic configuration were maintained across all three architectures. For nnU-Net, model development on the remaining cases followed the framework’s self-configuring cross-validation and training procedure. Model selection was based exclusively on validation performance according to the corresponding architecture-specific training strategy.

A separate Hybrid synthetic–clinical experiment was conducted to examine one-stage model development using combined synthetic and limited clinical data. In this configuration, the 120 synthetic cases were combined with the 10 clinical development cases, yielding a 130-case development pool that was divided into 104 training and 26 validation cases. The fixed 10-case clinical test cohort remained completely excluded from training and validation and was used only for evaluation. Models in the Hybrid experiment were trained from random initialization and did not provide initialization weights for the subsequent clinical fine-tuning or ensemble pathway.

For the sequential synthetic-to-clinical pathway described in Section 2.8, the Mixed-size synthetic configuration was selected as the source-domain initialization because it included both synthetic lesion-size categories, represented the broadest synthetic lesion distribution, and comprised the largest purely synthetic cohort evaluated in the study. This choice should not be interpreted as evidence that Mixed-size pretraining was superior to the size-specific configurations for clinical transfer, because alternative synthetic source configurations were not compared under the same clinical fine-tuning protocol.

### 2.8. Clinical Fine-Tuning

In the second experimental pathway, models initialized from the Mixed-size synthetic configuration were adapted to the clinical imaging domain using exclusively the 10-case clinical development cohort. The Mixed-size configuration served as the common source-domain initialization for all three architectures, as described in Section 2.7.

Clinical fine-tuning was performed using five-fold cross-validation within the development cohort, with identical fold assignments used across architectures. In each fold, eight clinical cases were used for fine-tuning and two cases for validation. Consequently, each development case served once as a validation case and was excluded from the corresponding fold’s training subset. This procedure generated out-of-fold predictions for all 10 clinical development cases, which were subsequently used for ensemble configuration as described in Section 2.9.

For nnU-Net and Attention U-Net, clinical fine-tuning employed a combined cross-entropy and Tversky loss. The Tversky parameters were set to α = 0.3 and β = 0.7, assigning greater weight to false-negative than false-positive voxels and thereby prioritizing tumor coverage during adaptation. The baseline 3D U-Net was fine-tuned using binary cross-entropy loss to preserve consistency with its original optimization strategy.

Model selection was performed exclusively using validation data within the clinical development folds. The independent 10-case clinical test cohort was not used for fine-tuning, model selection, threshold selection, or ensemble configuration.

### 2.9. Ensemble Development, Configuration, and Clinical Evaluation

Following clinical fine-tuning, a heterogeneous ensemble was constructed from the nnU-Net, Attention U-Net, and baseline 3D U-Net models. Ensemble development was performed exclusively using the 10-case clinical development cohort. As described in Section 2.8, five-fold cross-validation generated out-of-fold probability maps for all development cases, with identical fold assignments used across the three architectures. Each out-of-fold prediction was therefore obtained from a model that had not been trained on the corresponding case.

For each voxel, the probability maps produced by the three architectures were combined using weighted probability averaging. To systematically evaluate the ensemble configuration, 13 model-weighting schemes were assessed at decision thresholds of 0.3, 0.5, and 0.6, resulting in 39 candidate configurations. The evaluated schemes included individual models, pairwise combinations, equal-weight averaging, and alternative heterogeneous three-model weightings. Mean Dice across the clinical development cases was used as the primary comparison criterion.

Within the evaluated comparison grid, the weighting scheme assigning relative weights of 2:2:3 to nnU-Net, Attention U-Net, and 3D U-Net, respectively, together with a decision threshold of 0.3, achieved the highest mean development Dice and was retained as the ensemble configuration for clinical evaluation.

For evaluation on the independent clinical test cohort, the probability maps generated by the five fold-specific models were averaged within each architecture, yielding one test probability map per architecture and examination. These architecture-level probability maps were subsequently combined using the selected ensemble weighting scheme.

The stability of the selected configuration was further examined using 1000 case-level bootstrap resamples and leave-one-case-out reselection within the clinical development cohort. Additional sensitivity analyses evaluated an expanded range of weight–threshold combinations and alternative probability aggregation strategies; detailed results are provided in the Supplementary Material. All ensemble configuration and sensitivity analyses were conducted exclusively using clinical development predictions; the independent clinical test cohort was not used to compare or select ensemble weights, decision thresholds, or aggregation rules.

### 2.10. Evaluation Metrics and Statistical Analysis

Segmentation performance was evaluated using the Dice similarity coefficient (Dice), Intersection-over-Union (IoU), precision, and recall. Dice was defined as the primary endpoint because it directly quantifies spatial overlap between predicted and reference tumor masks and is widely used in medical image segmentation. IoU was included as a complementary overlap measure, whereas precision and recall were used to characterize false-positive and false-negative segmentation behavior, respectively. Recall refers here to voxel-level tumor recall rather than patient-level diagnostic sensitivity.

All metrics were calculated on a per-volume basis. For the synthetic and Hybrid dataset configurations, results were summarized across the corresponding held-out test cases using the mean and sample standard deviation, with 95% percentile-bootstrap confidence intervals additionally reported for mean Dice. For the independent clinical test cohort, descriptive statistics included the mean ± standard deviation, median and interquartile range, range, and 95% percentile-bootstrap confidence intervals. Confidence intervals were estimated using 10,000 case-level bootstrap resamples with replacement.

Statistical comparisons on the independent clinical test cohort were performed using paired methods because all segmentation approaches were evaluated on the same 10 DBT examinations. Given the limited cohort size and the bounded nature of the segmentation metrics, non-parametric paired analyses were used. For each metric, a Friedman test provided an omnibus assessment of differences among the four evaluated approaches: fine-tuned nnU-Net, Attention U-Net, 3D U-Net, and the heterogeneous ensemble.

Targeted pairwise comparisons between the ensemble and each individual architecture were additionally performed using two-sided Wilcoxon signed-rank tests. To account for the three ensemble-versus-model comparisons within each metric, *p*-values were adjusted using the Holm procedure. Matched-pairs rank-biserial correlation was reported as an effect-size measure. Rank-biserial correlation was signed according to the ensemble-minus-comparator difference; positive values favor the ensemble and negative values favor the individual model. Dice was treated as the primary inferential endpoint, whereas analyses of IoU, precision, and recall were considered secondary and exploratory. Statistical significance was defined as an adjusted *p* < 0.05.

Individual model probability maps were binarized using a threshold of 0.5. Ensemble predictions were generated using the weighting scheme and decision threshold determined from the clinical development cohort, as described in Section 2.9. The independent clinical test cohort was not used for ensemble configuration or threshold selection.

## 3. Results

### 3.1. Segmentation Performance Metrics Across Synthetic and Hybrid Configurations

Segmentation performance across the synthetic and Hybrid experimental configurations is summarized in Table 2. For the synthetic-only experiments, performance was evaluated on the corresponding held-out synthetic test sets, whereas the Hybrid models were evaluated on the fixed 10-case clinical test cohort.

Across the three synthetic configurations, nnU-Net achieved the highest mean Dice coefficient. In the Large Tumor configuration, mean Dice was 0.864 for nnU-Net, compared with 0.829 for the baseline 3D U-Net and 0.778 for Attention U-Net. A similar pattern was observed in the Mixed-size configuration, where nnU-Net achieved a mean Dice of 0.841, followed by 3D U-Net (0.726) and Attention U-Net (0.582).

Performance was lower for all architectures in the Small Tumor configuration. Mean Dice was 0.560 for nnU-Net, 0.367 for Attention U-Net, and 0.263 for 3D U-Net. The corresponding dispersion and wide confidence intervals should be interpreted in the context of the small held-out test set (*n* = 3). Collectively, these descriptive results indicate greater segmentation difficulty in the Small Tumor configuration than in the Large Tumor and Mixed-size configurations.

In the separate Hybrid synthetic–clinical experiment, evaluated on the 10 held-out clinical DBT examinations, mean Dice coefficients were 0.421 for Attention U-Net, 0.412 for the baseline 3D U-Net, and 0.353 for nnU-Net. These values were lower than those obtained by the corresponding models in the Large Tumor and Mixed-size synthetic test sets, illustrating the increased difficulty associated with evaluation in the clinical imaging domain. Because the Hybrid experiment combined synthetic and limited clinical development data in a one-stage training strategy, these results are interpreted as a separate synthetic–clinical comparator rather than as part of the sequential fine-tuning pathway evaluated in subsequent sections.

### 3.2. Clinical Fine-Tuning and Heterogeneous Ensemble Performance

Following clinical fine-tuning, the three individual architectures and the heterogeneous ensemble were evaluated on the fixed independent clinical test cohort comprising 10 DBT examinations. Table 3 summarizes the per-volume segmentation performance of the fine-tuned nnU-Net, Attention U-Net, and 3D U-Net models together with the ensemble.

The heterogeneous ensemble achieved the highest mean Dice coefficient (0.518 ± 0.263), compared with 0.482 ± 0.322 for nnU-Net, 0.372 ± 0.195 for Attention U-Net, and 0.367 ± 0.238 for 3D U-Net. The ensemble also yielded the highest mean IoU (0.384 ± 0.215) and recall (0.630 ± 0.315). Mean precision was 0.465 ± 0.259 for the ensemble, whereas nnU-Net and 3D U-Net each achieved a mean precision of approximately 0.50.

Considerable case-to-case variability was observed across all approaches, as reflected by the corresponding standard deviations, confidence intervals, and ranges reported in Table 3. Accordingly, the higher mean Dice observed for the ensemble should be interpreted descriptively at this stage; paired statistical comparisons between the ensemble and each individual architecture are reported separately in Section 3.4.

**Figure 3:**
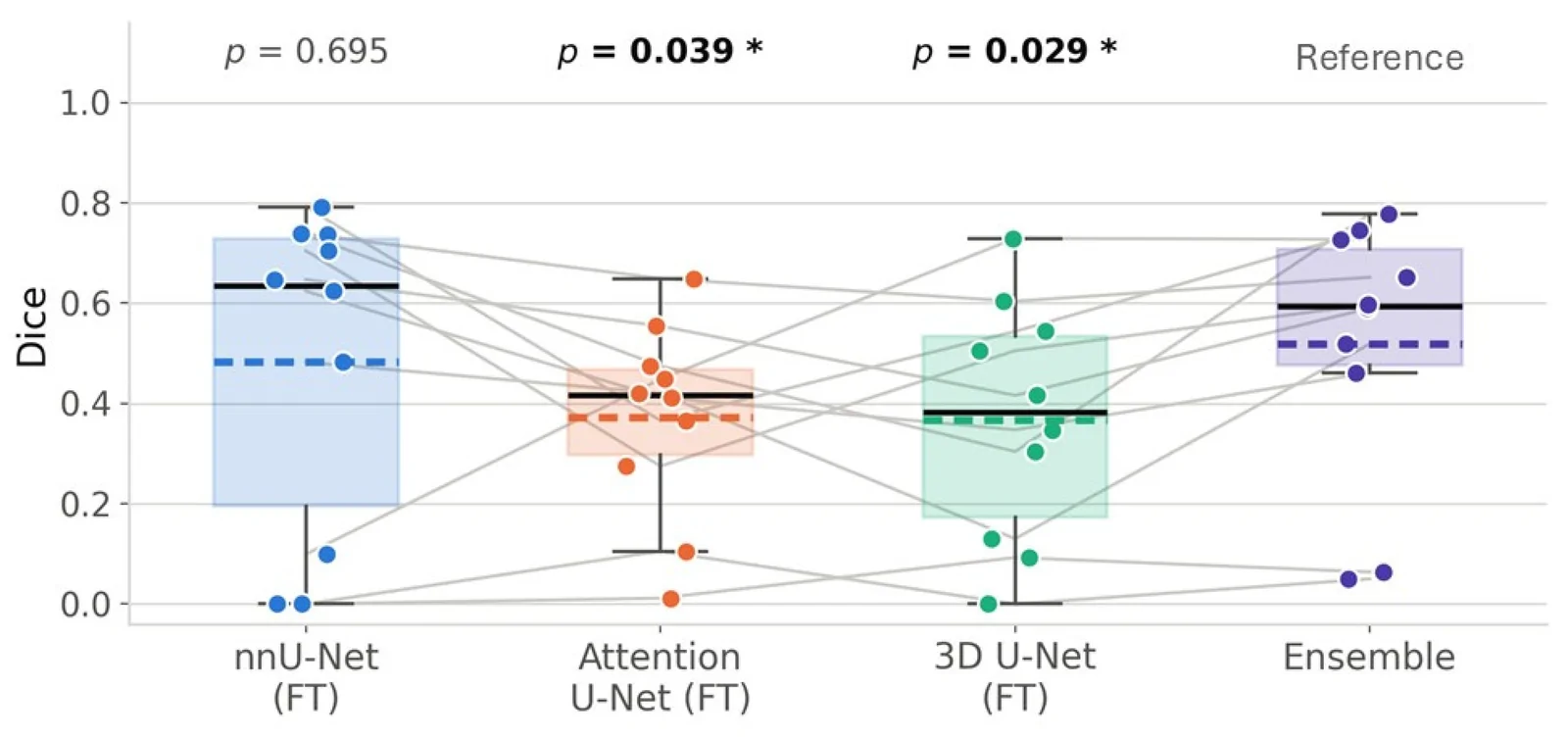
Paired case-level comparison of Dice performance on the independent clinical DBT test cohort. Dice coefficients are shown for the fine-tuned nnU-Net, Attention U-Net, 3D U-Net, and heterogeneous ensemble across the 10 held-out clinical examinations. Individual points represent clinical cases, and gray lines connect predictions obtained for the same examination across methods. Boxes represent the median and interquartile range, while dashed horizontal lines indicate the mean. Reported *p*-values correspond to two-sided Wilcoxon signed-rank comparisons between the ensemble and each individual architecture, adjusted using the Holm procedure across the three comparisons. Asterisks indicate adjusted *p* < 0.05; the ensemble serves as the reference.

To visualize the case-level variability underlying the summary statistics reported in Table 3, Figure 3 presents the paired Dice distributions for the 10 held-out clinical DBT examinations across the three fine-tuned architectures and the heterogeneous ensemble. Connecting individual examinations across methods preserves the paired structure of the evaluation and illustrates the variability in model performance between clinical cases.

The paired case-level distributions revealed substantial variability across the clinical examinations. Although the ensemble achieved the highest mean Dice, its difference relative to fine-tuned nnU-Net was not statistically significant (*p* = 0.695). In contrast, the ensemble showed significantly higher Dice than Attention U-Net (*p* = 0.039) and 3D U-Net (*p* = 0.029) after Holm correction. The complete paired statistical analysis, including effect sizes and secondary metrics, is presented in Section 3.4.

### 3.3. Ensemble Configuration and Stability Analysis

Ensemble configuration was evaluated exclusively using out-of-fold predictions from the 10-case clinical development cohort. Table 4 summarizes the ensemble ablation analysis across 39 candidate configurations, comprising 13 model-weighting schemes evaluated at decision thresholds of 0.3, 0.5, and 0.6. The evaluated configurations included individual models, pairwise combinations, equal-weight averaging, and alternative three-model weighting schemes.

The weighting scheme assigning relative weights of 2:2:3 to nnU-Net, Attention U-Net, and 3D U-Net, respectively, combined with a decision threshold of 0.3, achieved the highest mean Dice within the evaluated grid (0.582 ± 0.206; 95% CI, 0.458–0.698). The corresponding mean IoU, precision, and recall were 0.437, 0.562, and 0.696, respectively, with no development cases showing complete segmentation failure (Dice = 0). However, the performance margin relative to other highly ranked configurations was small, indicating that the selected configuration represented one of several similarly performing solutions rather than a sharply isolated optimum.

Stability analyses further evaluated the dependence of this selection on the limited development cohort. Across 1000 paired case-level bootstrap resamples, the 2:2:3 weighting scheme with a threshold of 0.3 was the most frequently selected configuration, ranking first in 52.6% of resamples and achieving a median rank of 1. The next most frequently selected configuration, 1:2:1 with a threshold of 0.6, was selected in 26.8% of resamples. Leave-one-case-out analysis retained the 2:2:3/0.3 configuration in 9 of 10 iterations; omission of one development case resulted in selection of the 1:2:1/0.6 configuration. These findings indicate relative stability of the selected configuration, although the selected parameters showed some dependence on which clinical development cases were included.

An expanded sensitivity analysis comprising 931 weight–threshold combinations further showed that the selected 2:2:3/0.3 configuration ranked third overall. The highest mean Dice in this expanded search was approximately 0.584, compared with approximately 0.582 for the selected configuration, corresponding to a difference of approximately 0.002. This broader analysis therefore supported the presence of a near-optimal performance region rather than a single uniquely optimal set of ensemble parameters. Alternative aggregation strategies, including unweighted probability averaging, log-odds aggregation, majority voting, maximum- and minimum-probability rules, and connected-component–based post-processing, did not provide a meaningful improvement over weighted probability averaging. Detailed sensitivity and alternative aggregation results are provided in the Supplementary Figure S1 and Table S1.

All ensemble configuration, stability, and sensitivity analyses were performed exclusively using predictions from the clinical development cohort. The independent clinical test cohort was not used to compare or select ensemble weights, decision thresholds, or aggregation strategies.

### 3.4. Paired Statistical Comparison on the Clinical Test Cohort

Paired statistical comparisons were performed across the 10 independent clinical test examinations because all four segmentation approaches were evaluated on the same cases. Table 5 summarizes the Friedman omnibus tests and the targeted Wilcoxon signed-rank comparisons between the heterogeneous ensemble and each individually fine-tuned architecture, with Holm adjustment applied to the three pairwise comparisons within each metric.

For the primary endpoint, Dice, the Friedman test indicated an overall difference among the four approaches (χ^2^ = 8.27, df = 3, *p* = 0.041). Pairwise analysis showed that the ensemble achieved significantly higher Dice than Attention U-Net (Holm-adjusted *p* = 0.039, rank-biserial r = +0.82) and 3D U-Net (Holm-adjusted *p* = 0.029, r = +0.89). In contrast, the difference between the ensemble and nnU-Net was not statistically significant (Holm-adjusted *p* = 0.695, r = −0.16). The ensemble achieved a higher Dice than nnU-Net in 5 of 10 cases, whereas nnU-Net achieved the higher Dice in the remaining 5 cases. These findings indicate that, although the ensemble yielded the highest mean Dice across the clinical cohort, its advantage over fine-tuned nnU-Net was not supported by the paired inferential analysis.

Analyses of IoU, precision, and recall were treated as secondary and exploratory outcomes. The corresponding omnibus and ensemble-versus-model comparisons are reported in Table 5. These analyses showed metric-dependent differences between approaches and should be interpreted cautiously given the limited number of clinical test cases and the exploratory status of these secondary endpoints.

Case-level analysis further demonstrated heterogeneous failure patterns across architectures. Complete segmentation failures (Dice = 0) occurred in 2 of 10 cases for nnU-Net and 1 of 10 cases for 3D U-Net, whereas no complete failures were observed for Attention U-Net or the ensemble. When Dice < 0.10 was used to characterize very poor overlap, such cases occurred in 3/10, 1/10, 2/10, and 2/10 examinations for nnU-Net, Attention U-Net, 3D U-Net, and the ensemble, respectively. These failure counts are descriptive and were not interpreted as evidence of statistically different failure rates. Case-level Dice values underlying the paired comparisons are provided in Supplementary Table S2.

### 3.5. Qualitative Assessment of Clinical Segmentation Performance

**Figure 4:**
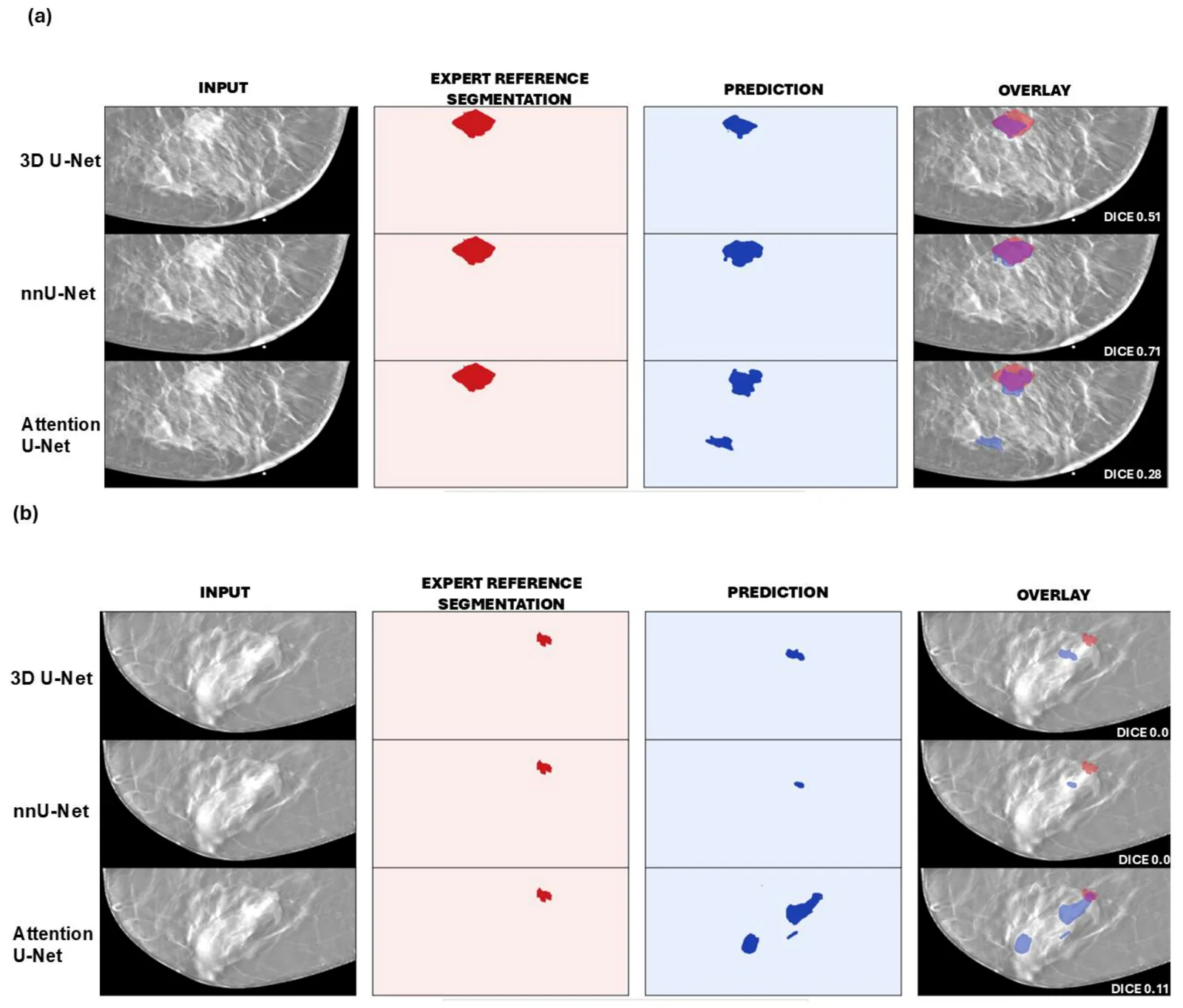
Qualitative comparison of individually fine-tuned segmentation models on the independent clinical DBT test cohort. Representative predictions from 3D U-Net, nnU-Net, and Attention U-Net are shown for two clinical examinations illustrating different levels of segmentation agreement. (**a**) Representative higher-overlap case. (**b**) Representative lower-overlap case. For each architecture, the columns show the input DBT slice, expert reference segmentation, predicted tumor mask, and corresponding prediction–reference overlay. The examples illustrate differences among architectures in lesion localization, segmentation completeness, boundary agreement, and false-positive behavior.

**Figure 5:**
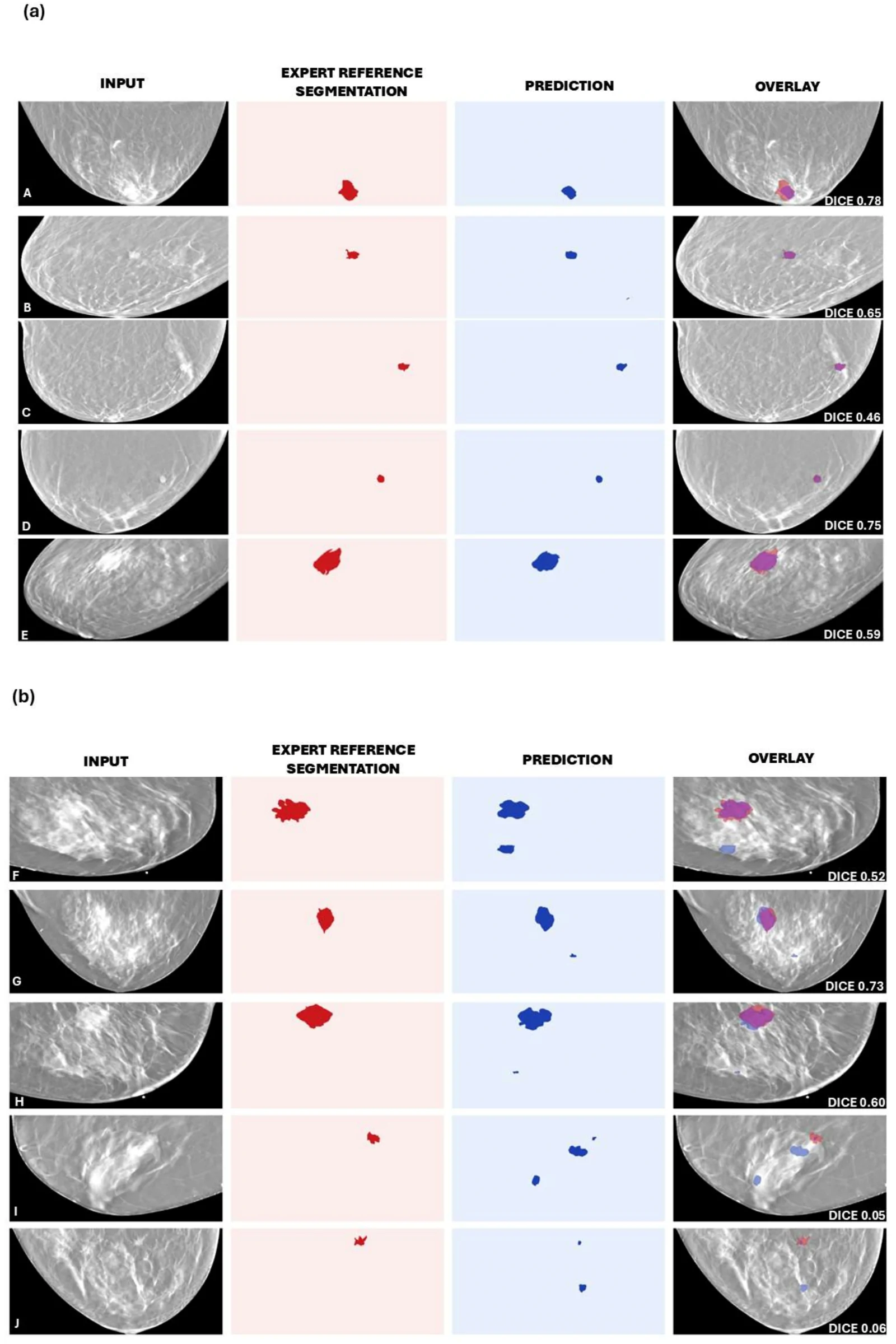
Qualitative segmentation results of the heterogeneous ensemble on the independent clinical DBT test cohort. Representative examples from the held-out clinical examinations are shown to illustrate variation in ensemble segmentation performance. For each case, the columns display the input DBT slice, expert reference segmentation, ensemble prediction, and corresponding overlay between prediction and reference. Panel (**a**) shows cases with relatively higher segmentation agreement (rows A–E), whereas panel (**b**) shows more challenging cases characterized by incomplete delineation (rows F,G), reduced prediction–reference overlap (row H), or false-positive segmentation (rows I,J). The examples illustrate the remaining variability in ensemble performance across clinical examinations despite the higher mean Dice, IoU, and recall observed for the ensemble.

Figure 4 and Figure 5 provide representative qualitative examples from the independent clinical test cohort. Figure 4 compares predictions from the individually fine-tuned nnU-Net, Attention U-Net, and 3D U-Net models, whereas Figure 5 illustrates the corresponding behavior of the heterogeneous ensemble across representative clinical cases. The examples were selected to illustrate both higher- and lower-overlap segmentation outcomes and to complement the quantitative and paired statistical analyses reported above.

Visual inspection revealed substantial case-dependent variability in segmentation behavior. In higher-performing cases, the predicted masks generally overlapped the reference lesion and reproduced its overall spatial extent, although differences in boundary delineation remained evident between architectures. In lower-performing cases, the observed qualitative error patterns consisted of incomplete lesion delineation, under-segmentation of portions of the reference mask, and false-positive segmentation in adjacent breast tissue. Individual architectures also exhibited occasional complete or near-complete segmentation failures, consistent with the case-level performance patterns described in Section 3.4.

The ensemble did not eliminate these errors but generally maintained lesion localization across the illustrated cases while showing variable agreement with the reference boundaries. These qualitative findings are therefore consistent with the quantitative results: ensemble integration improved mean performance relative to some individual architectures, but clinically relevant segmentation errors remained present and varied substantially between examinations.

## 4. Discussion

This proof-of-concept study investigated the feasibility of combining synthetic pretraining, limited clinical fine-tuning, and heterogeneous ensemble learning for volumetric breast tumor segmentation in digital breast tomosynthesis (DBT) under constrained annotation conditions. Four principal observations emerged from the analysis. First, volumetric segmentation using 15-slice full-field-of-view DBT inputs was feasible across the evaluated synthetic configurations, supporting the use of synthetic DBT data for controlled model development under limited clinical annotation availability. Second, segmentation performance varied according to the synthetic lesion-size configuration, with consistently lower performance observed in the Small Tumor dataset than in the Large Tumor and Mixed-size configurations. Third, performance decreased when models were evaluated in the clinical imaging domain, highlighting the persistent synthetic-to-clinical domain gap. Finally, the heterogeneous ensemble achieved the highest mean Dice on the independent clinical test cohort and significantly exceeded Attention U-Net and 3D U-Net, whereas its difference from fine-tuned nnU-Net was not statistically significant. Collectively, these findings support the methodological feasibility of synthetic-to-clinical volumetric DBT segmentation while emphasizing that ensemble integration may improve robustness relative to some individual architectures without establishing universal superiority over the strongest single-model approach.

A relevant methodological contribution of this work is the adoption of a full field-of-view volumetric segmentation framework. Most previous DBT segmentation studies have relied on lesion-centered regions of interest, cropped image patches, or slice-based analyses that simplify the segmentation task by restricting the search space and implicitly providing lesion localization [24,25,26,27,54]. In contrast, the present framework preserves the complete in-plane breast field of view within a 15-slice volumetric input and requires the models to localize and delineate the lesion without lesion-centered in-plane cropping. Although this increases task complexity, it more closely approximates the conditions of clinical image interpretation, in which lesion location is not provided a priori. Consequently, the segmentation performance reported in this study should be interpreted in the context of a more demanding localization-and-segmentation setting than approaches based on predefined lesion-centered inputs.

Lesion size was an important determinant of segmentation performance within the synthetic domain. Across all evaluated architectures, performance was consistently lower in the Small Tumor configuration than in the Large Tumor and Mixed-size configurations, indicating greater difficulty in segmenting lesions with limited volumetric extent. Similar challenges have been reported across medical imaging applications, where small-lesion segmentation is affected by severe class imbalance, reduced morphological information, and limited contextual cues for feature extraction [55,56,57,58]. In DBT, these difficulties may be further compounded by anisotropic spatial resolution, limited-angle acquisition, and reconstruction artifacts [19,20]. However, the performance of the Mixed-size configuration should not be attributed solely to lesion-size heterogeneity, because this dataset also contained more cases than either size-specific configuration. Thus, the observed results may reflect the combined effects of lesion-size distribution, sample size, and overall variability, which were not independently controlled in the present study.

A central challenge identified in this study was the limited generalization from synthetic to clinical DBT. Synthetic training provided the source-domain initialization used for clinical fine-tuning, but segmentation performance decreased when the models were evaluated on previously unseen clinical examinations, indicating a substantial synthetic-to-clinical domain gap. Although nnU-Net remained the strongest individual architecture after clinical fine-tuning, the magnitude of performance differences and case-level behavior varied between the synthetic and clinical domains. These findings indicate that synthetic-domain performance alone does not fully characterize target-domain behavior.

Despite the observed domain gap, synthetic DBT data remain valuable for controlled model development, reproducible experimentation, and source-domain pretraining when annotated clinical data are scarce. Physics-based simulation enables systematic control of lesion characteristics and imaging conditions while providing complete voxel-level reference segmentations, thereby supporting experiments that would be difficult to conduct using limited clinical cohorts alone. Nevertheless, synthetic datasets cannot fully reproduce the anatomical variability, biological heterogeneity, acquisition characteristics, reconstruction artifacts, and noise encountered in clinical imaging [31,32,35,36]. Accordingly, the findings of the present study support the use of synthetic DBT data as a complementary resource for model initialization and methodological evaluation rather than as a replacement for clinical imaging data.

While synthetic pretraining establishes a common source-domain starting point, architecture diversity may also influence model behavior following clinical adaptation. In the present study, the three fine-tuned architectures exhibited distinct case-level performance patterns on the independent clinical cohort, with no single model consistently providing the highest Dice across all examinations. These differences suggest that the architectures responded differently to individual clinical cases, creating the potential for heterogeneous prediction integration to reduce model-specific errors. The ensemble achieved the highest mean Dice across the clinical cohort and significantly exceeded Attention U-Net and 3D U-Net; however, its difference from fine-tuned nnU-Net was not statistically significant. Accordingly, the present findings support heterogeneous ensemble learning as a potentially useful strategy for integrating partially different prediction patterns, but they do not demonstrate universal superiority over the strongest individual architecture.

Previous studies have demonstrated the potential value of model diversity in medical image segmentation. Kamnitsas et al. [41] showed that combining independently optimized convolutional neural networks could improve robustness by reducing architecture-specific prediction variability. In breast imaging, Khaled et al. [42] reported improved breast lesion segmentation in DCE-MRI using U-Net ensembles, whereas Rahimpour et al. [43] demonstrated that ensemble selection across multiple networks could improve three-dimensional breast tumor segmentation performance. These studies support the broader rationale for integrating predictions from heterogeneous architectures rather than relying on a single model. In the present study, ensemble integration similarly yielded the highest mean Dice on the independent clinical cohort and significantly exceeded two of the three individual architectures. However, the absence of a statistically significant difference relative to fine-tuned nnU-Net indicates that the benefit of heterogeneous integration was not uniform across all comparisons. Thus, the present findings are consistent with prior evidence supporting ensemble-based robustness, while also emphasizing that the magnitude of benefit may depend on the constituent models and the characteristics of the evaluated cases.

Future improvements in synthetic-to-clinical reliability will likely require methodologies that address the domain gap more directly than ensemble integration alone. Approaches such as explicit domain adaptation, feature or representation alignment, self-supervised pretraining, and uncertainty-aware learning may help reduce discrepancies between simulated and clinical DBT data [35,36,40]. More recent segmentation paradigms, including transformer-based architectures for 3D medical image segmentation and foundation-model approaches adapted to volumetric medical imaging, also represent relevant directions for future investigation [44,45]. These strategies were not experimentally evaluated in the present study because the primary objective was to isolate the feasibility of a comparatively simple pipeline combining synthetic pretraining, limited supervised clinical fine-tuning, and heterogeneous ensemble learning under constrained annotation conditions. Future studies should therefore determine whether incorporating these more advanced adaptation and representation-learning strategies can provide additional gains in clinical generalization beyond those observed with the present framework.

The qualitative evaluation further complemented the quantitative findings by illustrating substantial case-dependent variability in segmentation behavior. In higher-performing examinations, the individual models and the ensemble generally localized the lesion and reproduced its overall spatial extent, although differences in boundary delineation remained evident. In lower-performing cases, the observed error patterns included incomplete lesion delineation, under-segmentation of portions of the reference mask, false-positive segmentation in adjacent breast tissue, and occasional complete or near-complete segmentation failures. No complete Dice = 0 failures were observed for the ensemble. These observations indicate that, while ensemble integration may reduce some architecture-specific errors, achieving accurate and consistent lesion delineation across heterogeneous clinical examinations remains a challenging task.

Beyond the specific application to DBT, the present work also has broader methodological implications for synthetic-to-clinical learning. Although nnU-Net remained the strongest individual architecture after clinical adaptation, the relative performance gaps among architectures and their case-level behavior differed between source and target domains. This indicates that source-domain performance alone should not be used as a surrogate for clinical transfer performance. Evaluating synthetic pretraining together with subsequent adaptation and independent clinical testing therefore provides a more informative characterization of generalization than synthetic-domain benchmarking alone.

### Limitations and Future Work

Several limitations should be acknowledged. First, the clinical component of the study comprised only 20 annotated DBT examinations, with 10 cases used for clinical development and 10 reserved for independent testing. Although paired non-parametric analyses and bootstrap confidence intervals were used to quantify uncertainty, the limited cohort size restricts statistical power, the precision of performance estimates, and the generalizability of the findings. Second, all reference segmentations were generated or reviewed by a single experienced breast-imaging expert using a standardized annotation protocol; therefore, inter-observer variability could not be assessed. Third, detailed clinical characteristics relevant to lesion stratification, including maximum tumor diameter, T stage, and neoadjuvant treatment history, were not available from the public clinical dataset. The selected clinical cases corresponded to single-lesion examinations, but more detailed characterization of multifocal or multicentric disease was unavailable. Finally, the clinical evaluation was restricted to a single publicly available dataset, and external validation using larger, independent, multi-institutional cohorts was not performed.

From a methodological perspective, the present study was intentionally designed to evaluate a focused synthetic-to-clinical pipeline based on three established volumetric convolutional architectures, limited supervised clinical adaptation, and heterogeneous ensemble integration. Accordingly, several extensions were beyond the scope of the current work. First, the models operated on standardized 15-slice volumetric inputs rather than the complete reconstructed DBT examination, although the full in-plane breast field of view was preserved and no lesion-centered cropping was applied. Second, explicit domain-adaptation techniques and more recent transformer-based or foundation-model approaches were not included, as the objective was to isolate the feasibility and behavior of the proposed synthetic pretraining–fine-tuning–ensemble framework under constrained annotation conditions. Third, ensemble configuration relied on the 10-case clinical development cohort; however, the additional bootstrap, leave-one-case-out, and expanded sensitivity analyses provided evidence that the selected weighting strategy was reasonably stable and belonged to a broader near-optimal performance region. These considerations define the current scope of the study and provide clear directions for extending the framework in larger and more diverse clinical cohorts.

Future research should build on the present framework by evaluating synthetic-to-clinical volumetric DBT segmentation in larger and more diverse clinical cohorts, including multi-institutional datasets and multi-reader reference annotations. Additional studies should also examine whether explicit domain-adaptation strategies, self-supervised representation learning, uncertainty-aware segmentation, and transformer- or foundation-model–based approaches can further improve clinical generalization when combined with synthetic pretraining. Particular attention should be given to identifying the conditions under which ensemble integration provides the greatest benefit and to evaluating whether the near-optimal parameter region observed in the present study remains stable across broader clinical populations. Such extensions would complement the current findings and help determine how the proposed synthetic-to-clinical learning framework can be generalized toward more robust volumetric DBT segmentation.

## 5. Conclusions

This proof-of-concept study investigated synthetic-to-clinical volumetric breast tumor segmentation in digital breast tomosynthesis under limited annotated data conditions. The findings support the use of synthetic DBT data as a valuable source-domain resource for developing volumetric segmentation models while also demonstrating the persistent performance gap encountered when models are transferred to clinical imaging. By combining synthetic model development, limited supervised clinical fine-tuning, and independent clinical evaluation, the proposed framework enabled systematic assessment of segmentation performance under realistic data-constrained conditions.

Heterogeneous ensemble integration achieved the highest mean Dice on the independent clinical test cohort and significantly exceeded Attention U-Net and 3D U-Net, although its difference from fine-tuned nnU-Net was not statistically significant. The ensemble configuration and sensitivity analyses further indicated that the selected weighting strategy belonged to a broader near-optimal performance region rather than representing a uniquely optimal solution. These findings suggest that integrating heterogeneous prediction patterns may provide additional robustness during synthetic-to-clinical transfer, while also emphasizing that the magnitude of this benefit depends on the constituent models and individual clinical cases.

Although the present results do not support immediate clinical deployment, this study provides a reproducible methodological framework for investigating synthetic-to-clinical learning in volumetric DBT segmentation when expert-annotated clinical data are limited. Future validation in larger and more diverse clinical cohorts, together with complementary adaptation and representation-learning strategies, will help determine the generalizability and clinical potential of this approach.

## Figures and Tables

**Table 1:** Methodological comparison of representative studies relevant to DBT lesion analysis and volumetric medical image segmentation.

Study	Modality/Task	Input Formulation	Model Paradigm	Data Strategy/Evaluation	Methodological Relevance
Buda et al. [21]	Clinical DBT; mass and architectural-distortion detection	Reconstructed DBT volumes with lesion annotations	Deep learning detection framework	Large public clinical DBT cohort with separate development and test partitions	Established a major public clinical DBT resource; detection rather than voxel-level tumor segmentation.
Fan et al. [24]	Clinical DBT; mass detection	2D DBT slices with cross-slice fusion	Faster R-CNN	Clinical DBT evaluation in patients with breast masses	Slice-wise detection with volumetric fusion rather than direct volumetric segmentation.
Fan et al. [25]	Clinical DBT; mass detection and segmentation	3D image patches recombined within the DBT volume	3D Mask R-CNN	Clinical DBT training and independent test cohorts	Demonstrated 3D contextual processing for DBT mass analysis using patch-based volumetric inputs.
Qasem et al. [26]	Clinical DBT; mass segmentation	Representative 2D lesion-containing slice	AMS-U-Net	Automatic segmentation in malignant clinical cases	Automated DBT mass segmentation using 2D rather than volumetric input.
Shia et al. [27]	DBT of breast surgical specimens; tumor-margin segmentation	DBT images of excised breast tissue	Deep learning segmentation	Clinical surgical-specimen cohort	Demonstrated DBT segmentation in a specimen setting distinct from diagnostic breast-volume segmentation.
Alfaro Vergara et al. [37]	Synthetic and clinical DBT; tumor segmentation	2D lesion ROIs	U-Net, FCN, DeepLabv3/DeepLabv3+	In silico ROI training with synthetic-clinical evaluation	Established proof-of-concept use of synthetic DBT for segmentation, but with 2D ROI-based inputs.
Hatamizadeh et al. [44]	3D medical image segmentation	Volumetric 3D inputs represented as patch sequences	UNETR; transformer encoder with U-shaped decoder	Validated on public multi-organ 3D segmentation datasets	Representative transformer-based volumetric segmentation paradigm; not DBT-specific.
Wang et al. [45]	General-purpose volumetric medical image segmentation	Promptable 3D medical volumes	SAM-Med3D foundation model	Large-scale multi-dataset training and evaluation across volumetric targets	Representative 3D foundation-model approach; not specifically validated for DBT tumor segmentation.
Present study	Synthetic-to-clinical DBT tumor segmentation	15-slice volumetric input preserving full in-plane breast field of view	3D U-Net, nnU-Net, Attention U-Net, heterogeneous ensemble	Synthetic development, limited clinical fine-tuning, independent clinical testing	Integrates synthetic volumetric training, limited clinical adaptation, ensemble learning, and paired clinical evaluation.

Note. DBT, digital breast tomosynthesis; ROI, region of interest; R-CNN, region-based convolutional neural network; FCN, fully convolutional network.

**Table 2:** Segmentation performance across synthetic and Hybrid experimental configurations.

Model	Dice	IoU	Precision	Recall	95% CI (Dice)
(a) Synthetic datasets—held-out synthetic test sets					
Large tumor dataset (*n* = 8 test volumes)					
nnU-Net	**0.864 ± 0.069**	0.766 ± 0.102	0.883 ± 0.123	0.861 ± 0.076	[0.816, 0.904]
3D U-Net (baseline)	0.829 ± 0.088	0.717 ± 0.122	0.841 ± 0.111	0.846 ± 0.147	[0.768, 0.882]
Attention U-Net	0.778 ± 0.088	0.643 ± 0.116	0.766 ± 0.129	0.812 ± 0.114	[0.719, 0.832]
Small tumor dataset (*n* = 3 test volumes)					
nnU-Net	**0.560 ± 0.463**	0.476 ± 0.405	0.674 ± 0.223	0.574 ± 0.489	[0.028, 0.872]
3D U-Net (baseline)	0.263 ± 0.390	0.198 ± 0.308	0.234 ± 0.316	0.332 ± 0.477	[0.007, 0.712]
Attention U-Net	0.367 ± 0.370	0.269 ± 0.297	0.305 ± 0.339	0.505 ± 0.443	[0.000, 0.740]
Mixed-size tumor dataset (*n* = 11 test volumes)					
nnU-Net	**0.841 ± 0.105**	0.737 ± 0.133	0.894 ± 0.070	0.808 ± 0.139	[0.774, 0.887]
3D U-Net (baseline)	0.726 ± 0.238	0.608 ± 0.229	0.728 ± 0.267	0.738 ± 0.213	[0.573, 0.833]
Attention U-Net	0.582 ± 0.190	0.433 ± 0.187	0.604 ± 0.278	0.646 ± 0.143	[0.473, 0.685]
(b) Hybrid synthetic–clinical dataset—held-out clinical cohort (*n* = 10)					
nnU-Net	0.353 ± 0.382	0.279 ± 0.310	0.586 ± 0.456	0.365 ± 0.394	[0.132, 0.581]
3D U-Net (baseline)	0.412 ± 0.294	0.298 ± 0.238	0.542 ± 0.331	0.473 ± 0.350	[0.237, 0.582]
Attention U-Net	**0.421 ± 0.252**	0.294 ± 0.195	0.410 ± 0.277	0.488 ± 0.312	[0.265, 0.560]

Note. Values are mean ± sample standard deviation. The 95% confidence interval corresponds to the percentile-bootstrap confidence interval for mean Dice. The highest mean Dice within each dataset configuration is shown in bold.

**Table 3:** Segmentation performance of clinically fine-tuned models and the heterogeneous ensemble on the independent clinical test cohort.

Model	Metric	Mean ± SD	Median [IQR]	95% CI	Range
nnU-Net	Dice	0.482 ± 0.322	0.635 [0.195–0.729]	[0.283, 0.659]	0.000–0.792
	IoU	0.367 ± 0.258	0.466 [0.119–0.574]	[0.210, 0.512]	0.000–0.655
	Precision	0.495 ± 0.351	0.535 [0.203–0.786]	[0.284, 0.696]	0.000–0.942
	Recall	0.497 ± 0.346	0.606 [0.189–0.740]	[0.286, 0.691]	0.000–0.906
Attention U-Net	Dice	0.372 ± 0.195	0.416 [0.298–0.468]	[0.255, 0.478]	0.012–0.649
	IoU	0.243 ± 0.142	0.263 [0.176–0.306]	[0.160, 0.324]	0.006–0.481
	Precision	0.291 ± 0.180	0.282 [0.211–0.405]	[0.186, 0.394]	0.008–0.616
	Recall	0.603 ± 0.275	0.621 [0.452–0.791]	[0.434, 0.756]	0.025–0.937
3D U-Net (baseline)	Dice	0.367 ± 0.238	0.382 [0.174–0.534]	[0.226, 0.508]	0.000–0.729
	IoU	0.249 ± 0.184	0.236 [0.097–0.365]	[0.143, 0.360]	0.000–0.574
	Precision	0.496 ± 0.234	0.589 [0.392–0.637]	[0.348, 0.622]	0.000–0.793
	Recall	0.338 ± 0.261	0.359 [0.113–0.469]	[0.188, 0.496]	0.000–0.808
Ensemble	Dice	**0.518** ± 0.263	0.594 [0.476–0.708]	[0.356, 0.661]	0.050–0.778
	IoU	**0.384** ± 0.215	0.422 [0.312–0.549]	[0.254, 0.504]	0.026–0.637
	Precision	0.465 ± 0.259	0.508 [0.352–0.603]	[0.309, 0.614]	0.034–0.878
	Recall	**0.630** ± 0.315	0.710 [0.570–0.860]	[0.434, 0.803]	0.053–0.919

Note. SD, standard deviation; IQR, interquartile range; CI, confidence interval; IoU, Intersection-over-Union. The highest mean Dice, IoU and Recall are shown in bold.

**Table 4:** Ensemble configuration ablation on the clinical development cohort.

Configuration	Weights	Thr.	Dice ± SD	95% CI	IoU	Prec.	Rec.	Zeros
Single model								
nnU-Net alone	1:0:0	0.3	0.476 ± 0.222	[0.337, 0.594]	0.335	0.469	0.561	0
	1:0:0	0.5	0.475 ± 0.225	[0.336, 0.595]	0.335	0.490	0.539	1
	1:0:0	0.6	0.472 ± 0.224	[0.332, 0.591]	0.332	0.502	0.521	1
Attention U-Net alone	0:1:0	0.3	0.411 ± 0.224	[0.281, 0.547]	0.283	0.323	0.675	0
	0:1:0	0.5	0.418 ± 0.226	[0.286, 0.554]	0.289	0.337	0.651	0
	0:1:0	0.6	0.421 ± 0.226	[0.289, 0.557]	0.291	0.343	0.640	0
3D U-Net alone	0:0:1	0.3	0.421 ± 0.236	[0.282, 0.556]	0.293	0.505	0.477	1
	0:0:1	0.5	0.414 ± 0.234	[0.277, 0.550]	0.287	0.514	0.462	1
	0:0:1	0.6	0.412 ± 0.233	[0.275, 0.547]	0.285	0.620	0.456	1
Pairwise								
nnU-Net + Attention	1:1:0	0.3	0.412 ± 0.226	[0.279, 0.542]	0.282	0.311	0.724	0
	1:1:0	0.5	0.491 ± 0.228	[0.352, 0.616]	0.351	0.449	0.601	1
	1:1:0	0.6	0.516 ± 0.213	[0.379, 0.624]	0.369	0.724	0.490	1
nnU-Net + 3D U-Net	1:0:1	0.3	0.547 ± 0.182	[0.437, 0.649]	0.396	0.497	0.703	0
	1:0:1	0.5	0.440 ± 0.217	[0.308, 0.559]	0.304	0.714	0.450	1
	1:0:1	0.6	0.301 ± 0.189	[0.189, 0.409]	0.190	0.753	0.309	1
Attention + 3D U-Net	0:1:1	0.3	0.466 ± 0.206	[0.346, 0.588]	0.326	0.373	0.755	0
	0:1:1	0.5	0.496 ± 0.171	[0.395, 0.595]	0.345	0.518	0.584	0
	0:1:1	0.6	0.398 ± 0.225	[0.262, 0.524]	0.269	0.718	0.376	1
Equal weights								
Equal weights	1:1:1	0.3	0.479 ± 0.190	[0.366, 0.588]	0.334	0.380	0.771	0
	1:1:1	0.5	0.520 ± 0.245	[0.371, 0.656]	0.383	0.658	0.564	0
	1:1:1	0.6	0.500 ± 0.241	[0.354, 0.635]	0.364	0.690	0.512	0
Selected								
**Selected scheme**	**2:2:3**	**0.3**	**0.582 ± 0.206**	**[0.458, 0.698]**	**0.437**	**0.562**	**0.696**	**0**
	2:2:3	0.5	0.504 ± 0.239	[0.360, 0.639]	0.367	0.659	0.537	0
	2:2:3	0.6	0.372 ± 0.223	[0.238, 0.496]	0.248	0.708	0.382	1
Alternative								
Alternative	2:1:1	0.3	0.516 ± 0.240	[0.367, 0.647]	0.378	0.471	0.648	0
	2:1:1	0.5	0.515 ± 0.249	[0.361, 0.649]	0.379	0.682	0.548	1
	2:1:1	0.6	0.474 ± 0.217	[0.339, 0.591]	0.332	0.709	0.467	1
Alternative	1:2:1	0.3	0.417 ± 0.224	[0.289, 0.552]	0.288	0.333	0.691	0
	1:2:1	0.5	0.494 ± 0.223	[0.360, 0.620]	0.353	0.464	0.606	0
	1:2:1	0.6	0.543 ± 0.235	[0.398, 0.670]	0.402	0.695	0.543	0
Alternative	1:1:2	0.3	0.578 ± 0.205	[0.456, 0.695]	0.433	0.572	0.681	0
	1:1:2	0.5	0.456 ± 0.224	[0.319, 0.579]	0.319	0.677	0.479	1
	1:1:2	0.6	0.365 ± 0.218	[0.234, 0.488]	0.242	0.698	0.379	1
Alternative	3:2:2	0.3	0.523 ± 0.226	[0.385, 0.647]	0.382	0.475	0.662	0
	3:2:2	0.5	0.521 ± 0.248	[0.370, 0.658]	0.385	0.663	0.565	0
	3:2:2	0.6	0.479 ± 0.222	[0.341, 0.599]	0.338	0.715	0.471	1
Alternative	2:3:2	0.3	0.428 ± 0.213	[0.309, 0.557]	0.295	0.347	0.697	0
	2:3:2	0.5	0.524 ± 0.245	[0.376, 0.661]	0.387	0.652	0.571	0
	2:3:2	0.6	0.537 ± 0.234	[0.393, 0.664]	0.396	0.704	0.532	0

Note. Thr., decision threshold; CI, confidence interval; IoU, Intersection-over-Union: Prec., precision; Rec., recall. The selected configuration is shown in bold.

**Table 5:** Paired statistical comparisons between the heterogeneous ensemble and individually fine-tuned models on the independent clinical test cohort.

Comparison	Median Diff.	95% CI	*p*	*p* (Holm)	r	Favors
Dice—Friedman χ^2^ = 8.27, df = 3, *p* = 0.041						
Ensemble vs. nnU-Net	−0.005	[−0.087, +0.053]	0.695	0.695	−0.16	5/10
Ensemble vs. Attention U-Net	+0.075	[+0.027, +0.303]	0.020	**0.039**	+0.82	9/10
Ensemble vs. 3D U-Net (baseline)	+0.103	[+0.024, +0.281]	0.010	**0.029**	+0.89	8/10
IoU—Friedman χ^2^ = 8.27, df = 3, *p* = 0.041						
Ensemble vs. nnU-Net	−0.017	[−0.102, +0.040]	0.492	0.492	−0.27	5/10
Ensemble vs. Attention U-Net	+0.062	[+0.019, +0.282]	0.010	**0.029**	+0.89	9/10
Ensemble vs. 3D U-Net (baseline)	+0.089	[+0.024, +0.221]	0.010	**0.029**	+0.89	8/10
Precision—Friedman χ^2^ = 4.76, df = 3, *p* = 0.190						
Ensemble vs. nnU-Net	−0.080	[−0.183, +0.057]	0.322	0.645	−0.38	4/10
Ensemble vs. Attention U-Net	+0.104	[+0.030, +0.302]	0.010	**0.029**	+0.89	8/10
Ensemble vs. 3D U-Net (baseline)	+0.022	[−0.113, +0.066]	1.000	1.000	−0.02	6/10
Recall—Friedman χ^2^ = 17.06, df = 3, *p* < 0.001						
Ensemble vs. nnU-Net	+0.075	[+0.046, +0.108]	0.064	0.129	+0.67	9/10
Ensemble vs. Attention U-Net	+0.026	[−0.031, +0.098]	0.432	0.432	+0.31	7/10
Ensemble vs. 3D U-Net (baseline)	+0.344	[+0.099, +0.442]	0.002	**0.006**	+1.00	10/10

Note. df, degrees of freedom; CI, confidence interval; IoU, Intersection-over-Union. Holm-adjusted *p* values < 0.05 are shown in bold. Dice was the primary endpoint; IoU, precision, and recall were secondary exploratory outcomes.

## Data Availability

The BreasTomo-Synth dataset V.1.1 used in the study is openly available through Zenodo at: https://doi.org/10.5281/zenodo.21450435 (last accessed on 2 September 2026). Breast Cancer Screening-Digital Breast Tomosynthesis dataset (BCS-DBT) (Version 5) is available through The Cancer Imaging Archive TCIA at: https://doi.org/10.7937/E4WT-CD02 (accessed on 3 January 2026). The T-SYNTH dataset is available through Hugging Face at: https://huggingface.co/datasets/didsr/tsynth (accessed on 1 February 2026). The source code and implementation details used for model training, fine-tuning, inference, and the experimental pipeline are publicly available at: https://github.com/GGP333/REPO_DBT_CA, (accessed on 1 September 2026).

## Supplementary Materials

The following supporting information can be downloaded at: https://www.mdpi.com/article/10.3390/diagnostics16183046/s1, Figure S1: Expanded sensitivity analysis of ensemble configuration on the clinical development cohort. Left: performance across decision thresholds. Center: heat map of weight–threshold combinations, with the selected configuration indicated. Right: contribution of individual ensemble members assessed by ablation, with bootstrap confidence intervals; Table S1: Comparison of alternative ensemble aggregation strategies on the clinical development cohort; Table S2: Case-level Dice coefficients for the individual fine-tuned models and heterogeneous ensemble on the independent clinical test cohort.

## Abbreviations

The following abbreviations are used in this manuscript:

DBT	Digital Breast Tomosynthesis
AI	Artificial Intelligence
ROIs	Regions of Interest
MRI	Magnetic Resonance Imaging
3D	Three-dimensional
