# Supplementary material for: Synthetic-to-Clinical Ensemble Learning for Volumetric Breast Tumor Segmentation in Digital Breast Tomosynthesis Under Limited Annotated Data
Source: Diagnostics (Basel). 2026 Sep 20;16(18):3046. doi: 10.3390/diagnostics16183046 (PMC13605567; doi:10.3390/diagnostics16183046)
Supplement: Supplementary file 1 [file diagnostics-16-03046-s001.zip › diagnostics-4453084-supplementary.pdf]

Supplementary Material

Synthetic-to-Clinical Ensemble Learning for Volumetric Breast Tumor Segmentation in Digital Breast Tomosynthesis Under Limited Annotated Data

Cristina Alfaro [1,2]*, Gabriel Guerra [3,4], Claudia Prieto [4,5], and Domingo Mery [4,6]

1 Department of Medical Technology, Facultad de Ciencias de la Salud, Universidad de Tarapacá, Arica, Chile.
2 Institute for Biological and Medical Engineering, Pontificia Universidad Católica de Chile, Santiago, Chile.
3 PhD Program in Health Sciences and Engineering, Universidad de Valparaíso, Valparaíso, Chile.
4 Millennium Institute for Intelligent Healthcare Engineering iHealth, Santiago, Chile.
5 Department of Electrical Engineering, School of Engineering, Pontificia Universidad Católica de Chile, Santiago, Chile.
6 Department of Computer Science, School of Engineering, Pontificia Universidad Católica de Chile, Santiago, Chile.

* Correspondence: calfarov@academicos.uta.cl

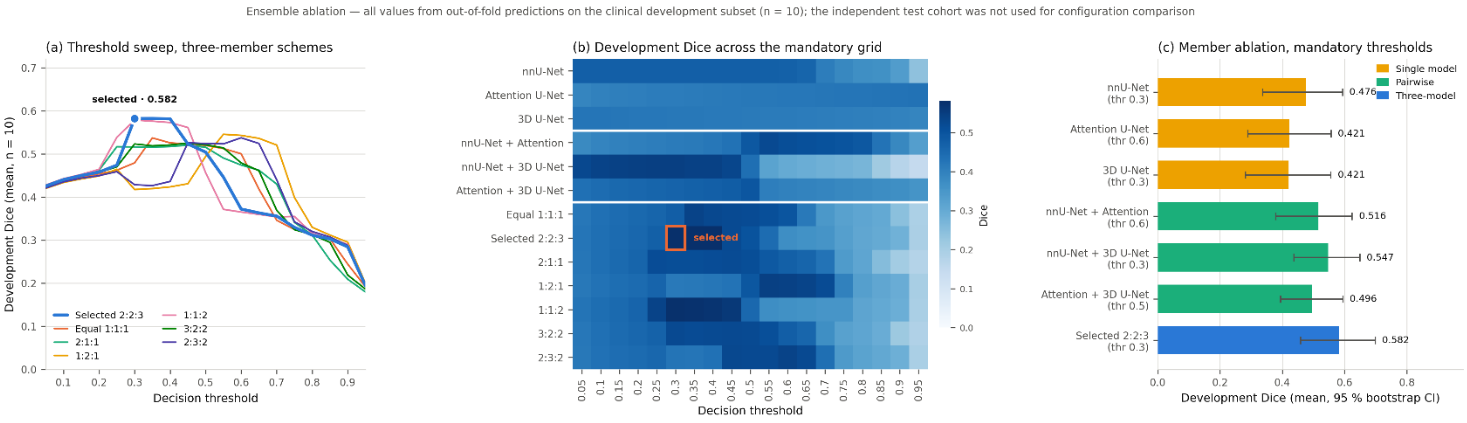

Figure S1. Expanded sensitivity analysis of ensemble configuration on the clinical development cohort. Left: performance across decision thresholds. Center: heat map of weight–threshold combinations, with the selected configuration indicated. Right: contribution of individual ensemble members assessed by ablation, with bootstrap confidence intervals.

Table S1. Comparison of alternative ensemble aggregation strategies on the clinical development cohort.

Aggregation rule	Threshold	Dice	Precision	Recall	Dice = 0
Mean log-odds	0.05	0.5821	0.592	0.675	0
Weighted mean (2:2:3)	0.35	0.5821	0.584	0.674	0
Weighted mean + largest connected component	0.3	0.5645	0.580	0.597	1
Unweighted mean (1:1:1)	0.35	0.5369	0.608	0.621	0
Majority vote (2/3 at 0.5)	N/A	0.5208	0.654	0.568	0
Voxel-wise maximum	0.9	0.4779	0.391	0.747	0
Voxel-wise minimum	0.05	0.3658	0.788	0.338	1

Note. Dice = 0 indicates the number of development cases with zero Dice overlap; N/A, not applicable.

Table S2. Case-level Dice coefficients for the individual fine-tuned models and heterogeneous ensemble on the independent clinical test cohort

Case	nnU-Net	Attention U-Net	3D U-Net	Ensemble	Ensemble − nnU-Net
005	0.737	0.475	0.304	0.778	+0.041
006	0.738	0.649	0.604	0.652	−0.087
007	0.624	0.412	0.347	0.462	−0.162
010	0.792	0.366	0.544	0.746	−0.046
013	0.647	0.554	0.416	0.590	−0.057
014	0.482	0.420	0.130	0.519	+0.036
**015**	**0.099**	**0.449**	**0.729**	**0.727**	**+0.628**
016	0.705	0.275	0.505	0.598	−0.107
019	0.000	0.105	0.000	0.050	+0.050
020	0.000	0.012	0.093	0.064	+0.064
Mean	0.482	0.372	0.367	0.518	+0.036

Note. Values are Dice coefficients. “Ensemble − nnU-Net” denotes the paired case-level Dice difference between the ensemble and nnU-Net.
